# The age of peak performance in Ironman triathlon: a cross-sectional and longitudinal data analysis

**DOI:** 10.1186/2046-7648-2-27

**Published:** 2013-09-01

**Authors:** Michael Stiefel, Beat Knechtle, Christoph Alexander Rüst, Thomas Rosemann, Romuald Lepers

**Affiliations:** 1 Institute of General Practice and for Health Services Research, University of Zurich, Pestalozzistrasse 24, Zurich 8091, Switzerland; 2 Gesundheitszentrum St. Gallen, Vadianstrasse 26, St. Gallen 9001, Switzerland; 3 INSERM U1093, Faculty of Sport Sciences - UFR STAPS, University of Burgundy, BP 27877, Dijon CEDEX 21078, France

**Keywords:** Endurance, Swimming, Cycling, Running

## Abstract

**Background:**

The aims of the present study were, firstly, to investigate in a cross-sectional analysis the age of peak Ironman performance within one calendar year in all qualifiers for Ironman Hawaii and Ironman Hawaii; secondly, to determine in a longitudinal analysis on a qualifier for Ironman Hawaii whether the age of peak Ironman performance and Ironman performance itself change across years; and thirdly, to determine the gender difference in performance.

**Methods:**

In a cross-sectional analysis, the age of the top ten finishers for all qualifier races for Ironman Hawaii and Ironman Hawaii was determined in 2010. For a longitudinal analysis, the age and the performance of the annual top ten female and male finishers in a qualifier for Ironman Hawaii was determined in Ironman Switzerland between 1995 and 2010.

**Results:**

In 19 of the 20 analyzed triathlons held in 2010, there was no difference in the age of peak Ironman performance between women and men (*p* > 0.05). The only difference in the age of peak Ironman performance between genders was in ‘Ironman Canada’ where men were older than women (*p* = 0.023). For all 20 races, the age of peak Ironman performance was 32.2 ± 1.5 years for men and 33.0 ± 1.6 years for women (*p* > 0.05). In Ironman Switzerland, there was no difference in the age of peak Ironman performance between genders for top ten women and men from 1995 to 2010 (*F* = 0.06, *p* = 0.8). The mean age of top ten women and men was 31.4 ± 1.7 and 31.5 ± 1.7 years (Cohen's *d* = 0.06), respectively. The gender difference in performance in the three disciplines and for overall race time decreased significantly across years. Men and women improved overall race times by approximately 1.2 and 4.2 min/year, respectively.

**Conclusions:**

Women and men peak at a similar age of 32–33 years in an Ironman triathlon with no gender difference. In a qualifier for Ironman Hawaii, the age of peak Ironman performance remained unchanged across years. In contrast, gender differences in performance in Ironman Switzerland decreased during the studied period, suggesting that elite female Ironman triathletes might still narrow the gender gap in the future.

## Background

The specific demands of a particular athletic challenge define the age of peak performance. Athletic tasks requiring strength, speed, and explosive power reach their peak in the early 20s, while tasks requiring endurance, acquired skills, and knowledge peak in the late 20s and early 30s [1]. Regarding different sports disciplines, swimmers achieve peak performance at 21 years, runners in track and field at 26 years [2], tennis players at 24 years [1], golf players at 31 years [1], and baseball players at 29 years [3]. In running, the age of peak performance increased with increasing length of the endurance performance [1,2,4-6], e.g., 23 years for 10,000 m [2], 30 years for a marathon [5], and 39 years for an ultra-marathon [5,6].

The potential gender difference in the age of peak performance has been sparsely investigated and controversially discussed. Schulz and Curnow reported that women achieved peak performance at a younger age compared to men in swimming, tennis, and running up to middle distances [1]. In marathon running, both sexes peak at a similar age [5], but the findings for running events longer than a marathon are not consistent [4,6,7]. In the 100-Mile Endurance Run in the USA, the top women were slightly older than the top men [6]. Ages of the top five men increased over the history of the race from around 30 years to generally being in the upper 30s. The average ages of the top five women seemed to be slightly higher than for men in the early 1980s and decreased through the decade; the ages of the top five women gradually increased since 1990 in a similar pattern as for men to reach the upper 30s in recent years. In the 100-km Lauf Biel in Switzerland, Europe, the top men were older than the top women [4]. Male winners with 38.2 ± 4.5 years were significantly older than female winners with 33.2±6.4 years. The mean age of the top ten female and male runners was 39.4 ± 2.3 and 40.4 ± 1.9 years, respectively. In the 78-km mountain ultra-marathon Swiss Alpine, the mean age of peak running times was unchanged between 1998 and 2011 at 34.4 ± 2.5 years for women and 33.9 ± 4.2 years for men [7].

In Ironman triathlon, Lepers and Maffiuletti showed that the best Ironman triathlon race times at the Ironman Hawaii were achieved by athletes between 25 and 39 years of age for both genders [8]. Further studies showed that master triathletes (i.e., triathletes older than 40 years of age) improved their Ironman triathlon performance over the last two decades, while younger triathletes seemed to have reached limits in their Ironman triathlon performance [9,10]. However, data on the age of peak performance for Ironman triathletes are lacking.

Gender differences in performance received considerable attention in recent decades. Several studies analyzed the differences in performance between the sexes in different disciplines, mainly running over different distances [5,11,12]. Over the past four decades, female participation in endurance events increased considerably, and during the same period, their endurance performances improved substantially [6,13]. These improvement rates exceeded those of men, leading to the hypothesis that gender difference would disappear in the future [14]. Moreover, it has been suggested that this difference should disappear in running as the distance increases, particularly beyond the marathon distance, because of advantages in fuel utilization and a greater fatigue resistance in women compared to men [15]. However, recent studies showed that gender difference in performance has plateaued [16], and longer distances were associated with greater gender differences in performance [12]. The 10%–15% gender difference in performance seems to be of biological origin as male endurance athletes possess larger aerobic capacity [17] and greater muscular strength [16].

The Ironman triathlon covering 3.8-km swimming, 180-km cycling, and 42-km running is one of the most challenging ultra-endurance events, and it represents an excellent model for examining the age of peak performance of endurance athletes and gender difference in endurance performance [8,18]. Lepers analyzed the performances of triathletes and gender differences at the Ironman Triathlon World Championship in Hawaii, USA, from 1981 to 2007 [18]. The gender difference in overall performance was approximately 13% and appeared smaller for swimming than for cycling or running. Over the last two decades, gender differences remained quite stable, except for running, where females tended to reduce the gap in the marathon part of the Ironman.

For Ironman triathletes, the exact age of peak performance is not known. In Ironman Hawaii, the fastest age groups in men were 30–34 and 35–39 years, and 25–29 and 30–34 years for women [8]. For triathletes competing in distances longer than the Ironman distance such as the Triple Iron ultra-triathlon (11.4-km swimming, 540-km cycling, and 126.6-km running) and the Deca Iron ultra-triathlon (38-km swimming, 1,800-km cycling, and 422-km running), the mean age of finishers was significantly higher for Deca Iron ultra-triathletes (41.3 ± 3.1 years) compared to Triple Iron ultra-triathletes (38.5 ± 3.3 years) [19]. For both ultra-distances, the fastest overall race times were achieved between 25 and 44 years. Considering existing results for the difference in the age of peak performance for ultra-endurance athletes [4,6], we may expect that female and male Ironman triathletes peak at a different age.

The aims of the present study were, firstly, to investigate in a cross-sectional analysis the age of peak Ironman performance within one calendar year in all qualifiers for Ironman Hawaii and Ironman Hawaii; secondly, to determine in a longitudinal analysis on a qualifier for Ironman Hawaii whether the age of peak Ironman performance and Ironman performance itself change across years; and thirdly, to determine gender difference in performance. Data from 20 Ironman races held in 2010 were analyzed cross sectionally, and data from Ironman Switzerland as a qualifier for Ironman Hawaii held between 1995 and 2010 were analyzed longitudinally. We hypothesized firstly that female Ironman triathletes would reach peak performance at a lower age compared to male Ironman triathletes. Secondly, we expected that the age of peak Ironman performance would remain unchanged across years, and thirdly, the gender difference in performance would be different between the three disciplines and change across years.

## Methods

For the longitudinal analysis of a potential change in the age of peak Ironman performance, the age of the annual top ten female and male finishers in a qualifier for Ironman Hawaii was determined in Ironman Switzerland between 1995 and 2010. For the cross-sectional analysis of the age of peak Ironman performance, the age of the top ten finishers for all qualifier races for Ironman Hawaii and Ironman Hawaii was determined in 2010. The study was approved by the Institutional Review Board of St. Gallen, Switzerland, with a waiver for the requirement of an informed consent given that the study involved the analysis of publicly available data.

### Longitudinal data analysis

From Ironman Switzerland, an official qualifier for Ironman Hawaii, the age of the annual top ten women and men was determined between 1995 and 2010. The data set from this study was obtained from the race website [20]. Ironman Switzerland is an important Ironman event in Europe because of the numerous qualifying places for the Ironman World Championship in Hawaii, the high participation rates, and its long history. It is the third oldest Ironman in Europe behind Ironman Europe (Germany) and Ironman Lanzarote (Spain). Since 1995, the event has been held in July each year in the city of Zurich. For the first 2 years, it was called Euroman and changed to Ironman Switzerland in 1997. Course records are 8 h 12 min for men (Oliver Bernhard in 2000) and 9 h 00 min for women (Karin Thürig in 2010). In 2010, more than 2,000 triathletes from more than 50 different countries started in Ironman Switzerland, and the female rate was 13.5%. Since 1995, almost 18,000 triathletes have competed in this race. Concerning qualifying places, Ironman Switzerland with 50 qualifying places for a start place at the Ironman World Championship in Ironman Hawaii is comparable with other qualifiers for Ironman Hawaii [21]. The course is held on a two-loop swim, a two-loop cycling course with 1,260 m of altitude gain, and a flat run course. The age at the time of competition and swimming, cycling, running, and overall race times of the top ten women and men in Ironman Switzerland were analyzed from 1995 to 2010. All times were converted to minutes. Age was calculated as the difference of the calendar year of the race minus the athlete's year of birth. The magnitude of gender difference was examined by calculating the percent difference for swimming, cycling, running, and overall race times of the top ten men versus the top ten women. The top ten time spread, i.e*.* the time difference between the winner and the tenth placer, was analyzed and expressed as a percentage of the winning performance for both women and men over the 1995–2010 period.

### Cross-sectional data analysis

A cross-sectional analysis of the age of peak performance was performed for all Ironman triathlons held in 2010. In 2010, a total of 22 Ironman triathlons, including 21 qualifier races and the Ironman World Championship, the Ironman Hawaii, were held. Complete data from the Ironman World Series 2010 were available for Ironman Hawaii and for all qualifier races held in 2010, with the exception of Memorial Hermann Ironman Texas, Ironman Wales, and Ford Ironman Cozumel [21]. From these three races, the complete rankings were not publicly available online, and the race directors were not able to provide us the data needed for analyses. Therefore, from 19 qualifiers and Ironman Hawaii, the exact ages of the top ten women and men were available. Since no information about age of the athletes was given for 2 out of the 21 qualifier races, these two races were excluded from analysis. From the other 20 competitions, the age of the overall top ten women and men at the time of competition was determined and analyzed.

### Statistical analysis

For the cross-sectional analysis, a Mann–Whitney test was used to compare the age of peak Ironman performance between women and men in each Ironman held in 2010. For the longitudinal analysis, linear regressions were used to estimate the changes in age and performances (split times, transition times, and overall race times) across the years 1995 to 2010. Pearson's correlation coefficients were used to assess the association between various variables. Effect size (Cohen's *d*) was defined as ‘small’ for 0 <*d* < 0.2, ‘medium’ for 0.2 <*d* < 0.5, and ‘large’ for 0.5 <*d* < 0.8. A one-way analysis of variance (ANOVA) was used to determine if the age of the top ten women and men differed over the studied period and to compare gender differences over the three disciplines. Tukey's *post hoc* analyses were used to test differences within the ANOVAs when appropriate. Statistical significance was accepted at *p* < 0.05 (Statsoft, Version 6.1, Statistica, Tulsa, OK, USA).

## Results

### Cross-sectional data analysis

Table 1 presents the age of the top ten women and men for each Ironman race held in 2010. In 19 of the 20 analyzed Ironman qualifiers held in 2010, there was no age difference between women and men (*p* > 0.05). The only age difference between the genders was seen in Ironman Canada where men were older than women (*p* = 0.023). For all 20 races (19 qualifier and Ironman Hawaii as the World Championship), the age of peak Ironman performance was 32.2 ± 1.5 years for men and 33.0 ± 1.6 years for women, respectively (*p* > 0.05). When Ironman Hawaii was excluded, the age of peak Ironman performance was 32.0 ± 1.4 years for men and 33.0 ± 1.6 years for women, respectively (*p* > 0.05).

**Table 1:** Mean (±SE) age of top ten women and men in all Ironman races in 2010

**Ironman race**	**Men**	**Women**
Ironman Arizona	31.5 ± 2.6	31.0 ± 3.4
Ironman Coeur d'Alene	32.8 ± 2.9	32.6 ± 3.7
Ironman Florida	32.0 ± 5.7	33.7 ± 6.4
Ironman Hawaii	34.2 ± 2.4	32.4 ± 3.2
Ironman Lake Placid	31.2 ± 4.4	31.0 ± 3.6
Ironman Louisville	31.7 ± 5.0	34.0 ± 4.5
Ironman St. George	30.0 ± 3.4	30.6 ± 4.2
Ironman Wisconsin	33.6 ± 5.4	30.8 ± 3.4
Ironman Germany	35.8 ± 4.8	32.3 ± 3.5
Ironman Australia	31.8 ± 5.4	32.1 ± 5.3
Ironman China	33.1 ± 5.5	35.7 ± 3.5
Ironman France	30.2 ± 3.7	32.3 ± 4.9
Ironman Regensburg	31.6 ± 4.1	34.6 ± 2.4
Ironman Switzerland	32.9 ± 5.7	34.8 ± 3.1
Ironman UK	32.0 ± 3.9	34.1 ± 4.4
Ironman Western Australia	33.3 ± 3.7	33.7 ± 5.8
Karnten Ironman Austria	31.3 ± 5.5	33.2 ± 5.8
Ironman New Zealand	30.4 ± 5.3	35.3 ± 5.2
Ironman South Africa	31.4 ± 4.6	31.2 ± 2.4
Ironman Canada	33.5 ± 4.0	29.5 ± 3.1*

*Significantly different (*p* = 0.023).

### Longitudinal data analysis

**Figure 1:**
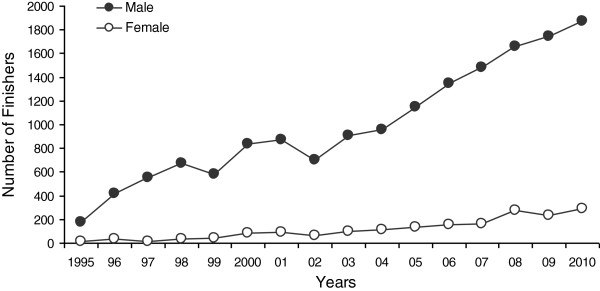
The number of female and male finishers in Ironman Switzerland from 1995 to 2010.

**Figure 2:**
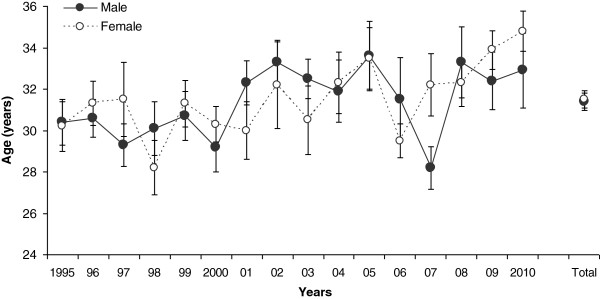
**Mean (±SE) age of top ten overall male and female finishers in Ironman Switzerland 1995–2010.** The years analyzed are pooled, and the mean age (±SE) of both genders is shown on the right side of the panel (Total).

From 1995 to 2010, there were 17,786 finishers in Ironman Switzerland (1,847 women and 15,939 men). The number of finishers each year over the history of the event is shown in Figure 1. There was a progressive rise in the number of finishers since 1995 for both sexes. Women accounted for 10%–14% of the field since 2001. From 1995 to 2010, there was no age difference between genders (*F* = 0.06, *p* = 0.8) for the top ten women and men (Figure 2). During this period, the mean age of the top ten women and men was 31.4 ± 1.7 years and 31.5 ± 1.7 years (Cohen's *d* = 0.06), respectively. The mean ages of male and female winners were 31.9 ± 3.5 years and 31.6 ± 3.7 years (Cohen's *d* = 0.08), respectively.

**Figure 3:**
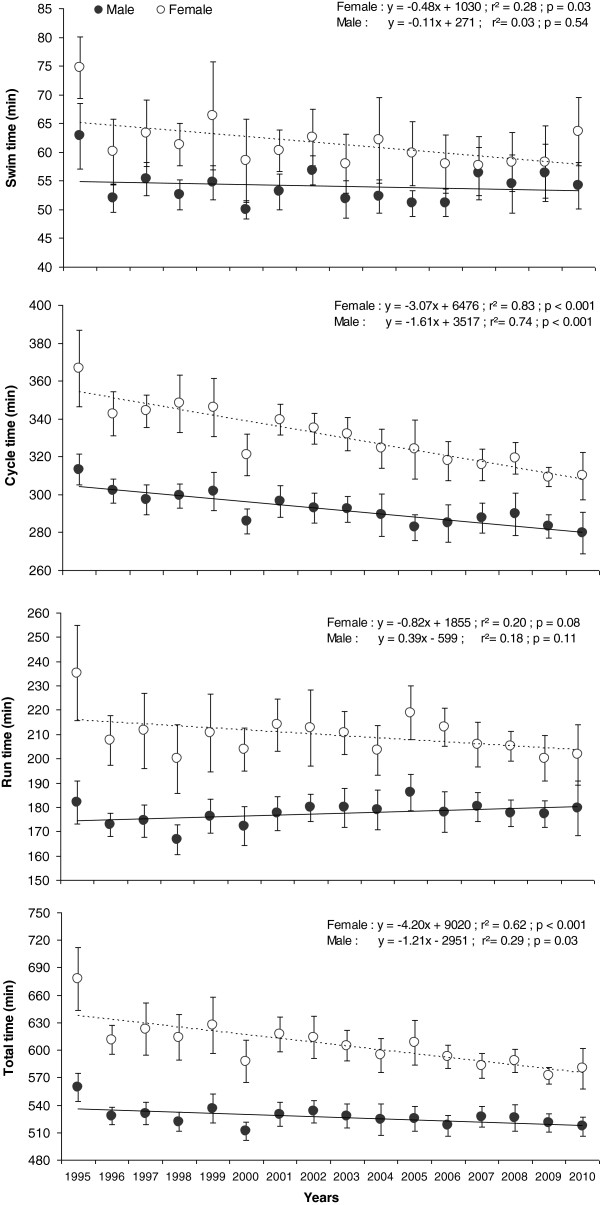
**Swimming, cycling, running, and overall race times.** In Ironman Switzerland for the top ten women and men from 1995 to 2010. Values are mean ± SD.

Figure 3 shows the historical performance trends of the top ten women and men between 1995 and 2010. Over this period, the mean swimming, cycling, running, and overall race times were 54.1 ± 3.1 min, 292.4 ± 8.9 min, 177.5 ± 4.4 min, and 527.3 ± 10.6 min for men, respectively, and 61.4 ± 4.3 min, 331.0 ± 16.0 min, 209.6 ± 8.7 min, and 605.9 ± 25.2 min for women, respectively. Regression lines are presented from 1995 to 2010 for the three disciplines and the overall race times for both genders. The gradient of the regression lines demonstrates that swimming and running performances have not significantly (*p* > 0.05) changed for both genders during the 1995–2010 period (Figure 3). In contrast, cycling times decreased significantly (*p* < 0.001) by approximately 3.1 min/year for women and by approximately 1.6 min/year for men, respectively. Men improved overall race times by approximately 1.2 min/year (*p* = 0.03) and women by approximately 4.2 min/year (*p* < 0.001), respectively. Regarding total transition time, ANOVA showed a main effect for gender. Total transition time in Ironman Switzerland was significantly longer for top ten women (4.37 ± 1.65 min) compared to top ten men (3.51 ± 0.98 min) during the studied period (*p* < 0.001).

**Figure 4:**
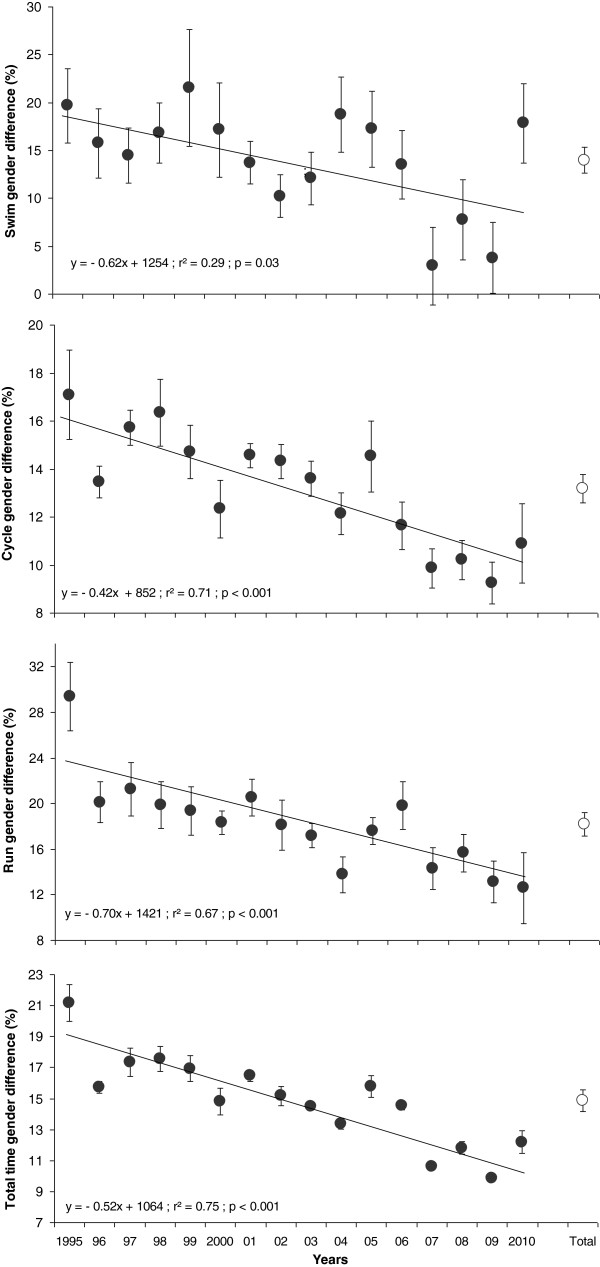
**Gender differences in swimming, cycling, running, and overall race times.** From 1995 to 2010 in Ironman Switzerland for the top ten women and men. The white circles on the right side represent the mean (±SE) gender difference for all the years (Total).

Figure 4 shows changes in gender difference in swimming, cycling, running, and overall race times between 1995 and 2010. Over this period, the difference in times decreased significantly in the three disciplines and for overall race time. The mean gender differences were 14.0 ± 5.4% for swimming, 13.2 ± 2.4% for cycling, 18.2 ± 4.1% for running, and 14.9 ± 2.9% for overall race time, respectively. The gender difference in running performances was significantly greater compared with swimming (*p* = 0.016) and cycling (*p* < 0.01).

**Figure 5:**
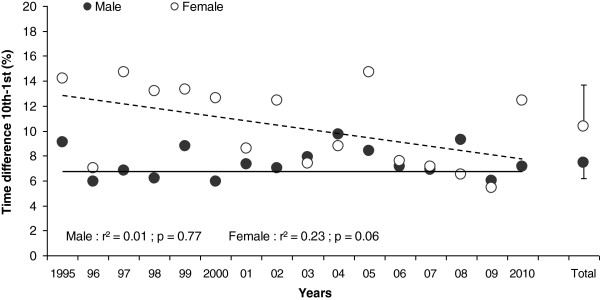
**Time difference between the winner and the tenth placer.** Expressed as a percentage of the winning time over the 1995–2010 period in Ironman Switzerland. The years analyzed are pooled, and the mean value (±SD) for both genders is shown on the right side of the panel (Total).

The relative time difference between the winner and the tenth placer for both women and men is shown in Figure 5. During the studied period, the tenth–first difference remained stable for men and decreased slightly for women. The mean tenth–first difference was 7.5 ± 1.3% for men and 10.4 ± 3.3% for women, respectively.

## Discussion

The aims of this study were, firstly, to investigate the age of peak Ironman performance in a longitudinal and cross-sectional analysis; secondly, to analyze gender difference in the age of peak performance and race performance in Ironman Switzerland from 1995 to 2010 as a qualifier for Ironman Hawaii; and thirdly, to determine gender difference in Ironman performance. The main findings were as follows: (1) the age of peak Ironman performance was not different between women and men at 32–33 years, (2) the age of peak Ironman performance showed no change across years although Ironman race times became faster for both women and men across years, and (3) the cycling times and overall race times decreased whereas the swimming and running times showed no changes.

### Age of peak ironman performance

Previous studies regarding the age of peak endurance performance showed that the age of peak performance was relatively constant over centuries because of the large role played by biological factors [1,2]. Data about the age of peak endurance performance can be useful in order to gain more detailed information about the physical development of humans [1] and to plan an athletic career. However, the potential gender difference in the age of peak endurance performance has been sparsely investigated and controversially discussed [1,4-6]. The present data showed that both male and female Ironman triathletes reached peak Ironman performance at the same age of around 32–33 years. Even if the Ironman triathlon is young in the field of ultra-endurance events with the first Ironman taking place in Hawaii in 1978 [18], and Ironman triathletes started with triathlon later in their life coming from a variety of sporting backgrounds with most from running, followed by swimming and cycling [22], the finding of no gender difference in the age of peak performance was constant over the 16-year period analyzed in Ironman Switzerland and confirmed by the cross-sectional analysis of all Ironman triathlons held in 2010. Furthermore, this is in agreement with studies on marathon runners. In marathon running, both genders peak at a similar age of 29–30 years [2,5]. Up to now, only age group Ironman performances have been analyzed, with the best performances reached between 25 and 39 years for both genders [8]. In marathon running, the fastest times were achieved by athletes in the age groups between 20 and 39 years [13,23], while ultra-runners seemed older, with the fastest age groups between 30 and 49 years [4,6,11].

### Gender difference in overall performance

An important finding was that despite the unchanged age in peak Ironman performance, both women and men improved Ironman performance across years in Ironman Switzerland with a decrease in gender difference. From the beginning in 1995 to 2010, gender difference in overall race time decreased significantly, attended by an increasing female participation in Ironman Switzerland. While women improved overall race time by approximately 4 min annually, men improved by approximately 1 min annually over the last 16 years. In contrast, it has been shown that the gender difference in overall race times remained stable over the last two decades at the Ironman World Championship in Hawaii [18].

The gender difference in overall race time of 15% for the overall top ten finishers in 2010 in Ironman Switzerland was slightly greater compared to the 13% in Ironman Hawaii (Table 2). This smaller gender difference in Ironman Hawaii is presumably because of the higher proportion of top athletes since Ironman Hawaii is held as the Ironman World Championship [21]. The top ten time spreads, i.e., the difference between the winner and the tenth placer is used to measure the extent of the highest level of competition [6,18]. The top ten time spreads for the overall top ten finishers in 2010 were less in Ironman Hawaii compared to Ironman Switzerland for both genders, but especially for women (Table 2). According to the slightly decreasing time spreads for female triathletes in Ironman Switzerland, the gender difference in overall race time is likely to come closer to those of Ironman Hawaii in the future.

**Table 2:** Gender difference (%) in Ironman triathlon performances and in time spreads

	**Gender difference (%)**	
	**Ironman Hawaii**	**Ironman Switzerland**
Performance		
Swim split	9.8 ± 2.9	14.0 ± 5.4
Cycling split	12.7 ± 2.0	13.2 ± 2.4
Running split	13.3 ± 3.1	18.2 ± 4.1
Total race time	12.6 ± 1.3	14.9 ± 2.9
Time spreads (first versus tenth)		
Men	4.8 ± 1.3	7.5 ± 1.3
Women	7.1 ± 2.3	10.4 ± 3.3

Data from Ironman Hawaii [18] versus Ironman Switzerland for the overall top ten athletes in 2010.

Gender differences in physiological characteristics are likely to be the main factor for this gender difference in overall race time. Men have larger skeletal muscle mass [24], correlating with greater muscular strength [16], larger aerobic capacity [17], and lower relative body fatness [25-27] compared to women. Low body fat is an important predictor variable for overall race time in an Ironman triathlon [28]. Low body fat was associated with faster race times in male Ironman triathletes [28], while in female triathletes, anthropometric characteristics such as body fat seemed not to be related to Ironman race time [25,27]. However, women retain 7%–9% more percent body fat compared to men [25,26], which is likely to be an advantage for men. Moreover, men probably have an advantage in muscular strength and aerobic capacity relative to the whole body mass [29]. Aerobic capacity is proportional to skeletal muscle mass, and both are strongly related to physical fitness [17]. Skeletal muscle mass is approximately 8% higher in male Ironman triathletes compared to female athletes, with 41.0 kg skeletal muscle mass in male versus 28.0 kg in female Ironman triathletes [17]. Aerobic capacity is approximately 14% higher in male Ironman triathletes, e.g., VO_2_peak is 61.3 versus 52.8 ml/kg/min in men and women, respectively [17].

### Gender difference in swim split performance

Although the gender difference in swimming decreased over the last 16 years in Ironman Switzerland, the triathletes' swimming performance showed no changes. On the other hand, in Ironman Hawaii, the gender difference in swimming remained very similar over the last two decades [18]. In Ironman Hawaii, the mean gender difference in swimming was approximately 10%, while it was approximately 14% in Ironman Switzerland. The 10% difference in Ironman Hawaii is in agreement with other studies concerning gender difference in swimming performance [30,31]. However, there are two main differences between the swim split in Ironman Hawaii and in Ironman Switzerland. Firstly, it is prohibited to wear a wet suit in Ironman Hawaii because of the warm ocean water temperatures. However, athletes were allowed to wear a wet suit in Ironman Switzerland every year. Secondly, the swim in Ironman Hawaii is held in the ocean with salt water in contrast to Ironman Switzerland where the swim takes place in fresh water in Lake Zurich. These two factors are likely to explain the difference between the 14% gender gap in Ironman Switzerland and the 10% gap in Ironman Hawaii. It is well known that female triathletes have more body fat [25,26]. Wet suits increase swimming performance by increasing buoyancy where lean subjects, especially, may benefit more from wearing wet suits than fatter subjects [32]. On the other hand, women with more body fat may profit more from the denser salt water in the ocean [18,33]. Another reason for the greater gender difference in Ironman Switzerland might be the aforementioned lower proportion of top female triathletes.

Wearing a wet suit seems to improve swimming performance and cycling performance. Wearing a wet suit while swimming leads to a significantly lower swimming cadence (-14%), significantly lower heart rate (-11%), and significantly lower lactate values (-47%) compared to swimming without a wet suit [34]. Moreover, cycling efficiency in a triathlon was significantly higher (+12.1%) after swimming with a wet suit compared to swimming without a wet suit [34]. In an Ironman triathlon where athletes can wear a wet suit in the swim split, athletes without the background as a swimmer may profit from wearing a wet suit and achieve faster swim times. The background aspect of the triathlete also seems to be of importance. It has been reported that many triathletes were competitive swimmers [35]. Regarding recreational Ironman triathletes competing in a qualifier for Ironman Hawaii such as the Ironman Lanzarote, 28% of the athletes had a background as a runner, 14% as a swimmer, and 13% as a cyclist [22]. However, for elite Ironman triathletes competing in Ironman Hawaii, the history of the athletes has not been investigated yet. Athletes with a background as a swimmer may swim fast in open water in Ironman Hawaii independent of whether they wear a wet suit or not since they are used as swimmers to swim fast in contrast to a weak swimmer. It has been shown that wearing a wet suit improves swim performance more in inefficient swimmers with low buoyancy when swimming at low speeds [36].

### Gender difference in cycling split performance

The annual top ten women and men improved cycling performance in Ironman Switzerland since 1995. Because of the greater improvement in women, the gender difference for the overall top ten finishers in 2010 in cycling has almost reached the level of Ironman Hawaii (Table 2). The mean gender difference in the 180-km cycling time trial is approximately 13%. Both cycling courses in Ironman Hawaii and Ironman Switzerland are relatively flat courses. In Ironman Hawaii, the altitude to climb is approximately 1,400 m, and in Ironman Switzerland, it is approximately 1,260 m. A comparison with other road cycling races is difficult because in these races the distances for women are shorter than those for men, and the races are generally held over shorter distances. For example, the pace difference between the first man and the first woman in the UCI Road World Championships in a time trial in 2010 held in Melbourne, Australia, was 11.4% (47.0 km/h versus 41.7 km/h). This 11.4% gender gap is likely to be underestimated because the distance for men was 45.8 km, but 22.8 km for women [37]. In track cycling, the gender difference is approximately 11% in race distances between 200 and 1,000 m [38]. Since this difference in cycling in Ironman Hawaii has increased over the last two decades [8], but decreased in Ironman Switzerland, both gender differences in cycling have approached approximately 13%. Thus, we conclude that the gender difference in cycling in Ironman triathlon is at approximately 13%. Moreover, this gender difference in performance of 13% is close to the 11% and above in other cycling races. The main reason for this 11%–13% gender difference in cycling performance is the greater muscle mass of the lower extremities in men [29].

Both women and men improved performance in the cycling split, but neither in the swimming nor in the running split. The improvement in the cycling split might explain the improvement in overall race time. We assume that Ironman triathletes invested more time in the preparation to improve in those split disciplines with more importance for the race. In an Ironman triathlon, the 3.8-km swim split accounts for only 1.7% of the total distance, whereas the 180-km cycling accounts for 79.6% and the marathon for 18.7% of the total race distance of 226 km. Recreational male Ironman triathletes invested 14.8 ± 3.2 h of training per week, and recreational female Ironman triathletes, 13.9 ± 3.4 h [26]. In men, swimming accounted for 2.5 h (16.9%), cycling for 8 h (54.0%), and running for 4 h (27.1%). Female Ironman triathletes invested 2.4 h in swimming (17.3%), 7.5 h in cycling (54.0%), and 4 h in running (28.8%).

Cycling performance can be improved by improvements in training and nutrition and alterations in equipment and seating position [39]. Factors such as aerodynamic accessories can modify the power demand in cycling [40]. Between 1995 and 2010, athletes may have improved both training and nutrition. Additionally, the bicycles and the aerodynamic accessories might have changed across years to improve cycling performance. All these improvements may have contributed to improved cycling performance and in the end an improved overall performance.

### Gender difference in running split performance

Running, along with cycling, is the most important discipline for a fast overall race time in a long-distance triathlon [41]. Women could have reduced the gender difference in marathon performance over the last 16 years in Ironman Switzerland, but there was no significant advance in running performance in either gender. On the other hand, women improved running performance by 3.8% per decade in Ironman Hawaii, with a relative improvement in running of 2.8% per decade for the top men [18]. The gender difference in running for the overall top ten finishers in 2010 in Ironman Switzerland was unexpectedly higher than that in Ironman Hawaii with approximately 18% in Ironman Switzerland and approximately 13% in Ironman Hawaii (Table 2). The gender difference in running in Ironman Hawaii is in line with the gender difference in top running events. Coast et al. compared the world's best running performances in distances between 100 m and 200 km [12]. The average gender difference was at 12.4% where longer distances were associated with greater gender differences [12]. In addition, the gender difference in performance in elite marathoners was approximately 12% [5], and the gender difference between the top five women and men in the Western States 100-Mile Endurance Run in 2007 was 14% [6].

However, the gender difference in running seemed to be higher if we focus not only on the top races but consider a wider range. Leyk et al. reported a 20% gap between the top ten male and female marathoners in 69 marathons in Germany [23]. The gender difference in the 100-km Lauf Biel was approximately 22% [4]. Hoffman reported that the fastest women were approximately 20% slower than the fastest men in all 161-km ultra-marathon competitions held in North America [11]. The difference of 14% in the gap in the Western States 100-Mile Endurance Run and approximately 20% in the other races is because the best times ever were achieved in the Western States 100-Mile Endurance Run, which is the oldest and most recognized 161-km running event [6,11]. We assume the same situation in Ironman triathlons with a smaller gender difference in Ironman Hawaii because this race is the Ironman Triathlon World Championship. These facts might be due to the lower proportion of top athletes, especially women, in Ironman Switzerland. In conclusion, we assume that the gender difference in running averages approximately 12%–14% in the premium running races and goes up to approximately 20% in a wider range of races. The effect of the smaller gender difference in top events compared with a wider range of events seems to be specific to running. Lepers and Maffiuletti compared the top ten amateur triathletes from nine age groups in Ironman Hawaii [8]. The difference was approximately 12% in swimming, approximately 15% in cycling, and approximately 18% in running for the 90 athletes of both sexes. There was a difference of approximately 2% in swimming and cycling compared to elite triathletes [18], while the difference in running was approximately 5% higher for the 90 amateur triathletes. In swimming, women may profit from their better economy compared to men [42]. In cycling, the muscular advantage of men is proportional to skeletal muscle mass, and it is even greater in running [29], where running is the only weight bearing activity where greater body fat is a limitation [25,26]. These are likely to be the reasons for the higher gender difference in running compared with cycling and swimming in a qualifying event for Ironman Hawaii. We assume that only the world's best women can overcome this disadvantage in running and that the top women in Ironman Switzerland have the best chance of improving their overall race time by improving their running performance.

### Methodological considerations

A limitation of the present study is that only split times and overall race times, age, and gender of the Ironman triathletes were analyzed. Other performance-related factors such as years of training and training volume [25,26], pre-race experience [25,43], metabolic factors, technical constraints, performance-enhancing drugs [18], and anthropometry [25-28,41] were not included. Regarding training and pre-race experience, training volume, cycling distance during training, speed in running during training, and both personal best marathon time and personal best time in an Olympic distance triathlon, were related to Ironman race time [22,25,26,43,44]. Furthermore, ultra-endurance performances such as an Ironman performance are influenced by weather conditions such as temperature, wind direction and velocity, rain, and water temperature [45]. The cycling performance depends on wind and weather conditions [18]. All these facts may influence performances, and they may limit comparison over the years. As the conditions in Ironman competitions are the same for every athlete, they do not influence the differences between the athletes, and there is no cause to assume that these facts influence the gender difference [18]. Concerning the difference between Ironman Switzerland and Ironman Hawaii, it should be mentioned that the weather conditions are different. Hawaii's climate is tropical in October when the Ironman takes place, i.e., approximately 30°C and approximately 70% humidity [46]. On the other hand, the climate in July is colder in Zurich, i.e., 20°C–25°C with humidity of approximately 70%. Wegelin and Hoffman reported that female performances were less affected by warm water than male performance [45]. The possibility of wearing a wet suit and the fresh water in Ironman Switzerland differs from Hawaii, while the cycling and running courses do not seem to be very different.

## Conclusions

The present findings suggested that the age of peak Ironman performance was similar for both women and men at around 32–33 years since there was no gender difference in the age of peak performance in all Ironman triathlons held in 2010 and over a 16-year period of Ironman Switzerland. The gender difference in performance in the three disciplines and for overall race time decreased significantly over the 1995–2010 period in Ironman Switzerland; however, gender differences in performance appeared greater than those observed in Ironman Hawaii, especially in running. This finding suggests that female Ironman triathletes participating in a qualifier for the World Championship Ironman Triathlon might be able to narrow the gap in the future. Future studies need to investigate the age of peak Ironman performance in the Ironman World Championship, the Ironman Hawaii.

## Abbreviations

VO2peak: Maximum oxygen uptake; UCI: International Cycling Union.

## Competing interests

The authors declare that they have no competing interests.

## Authors’ contributions

MS wrote the manuscript with the help of BK. BK collected the data. CAR and RL performed the statistical analyses. TR participated in the design and coordination and helped draft the manuscript. All authors read and approved the final manuscript.
